# The Role of Hematocrit Levels in Diagnosing the Severity of Acute Pancreatitis: A Cross-Sectional Study at a Tertiary Care Center in Nepal

**DOI:** 10.7759/cureus.68527

**Published:** 2024-09-03

**Authors:** Subodh Kumar Bidari, Milan Dhungana, Ram Chandra Panthi, Kushal Raj Joshi, Ritika Shrestha, Dinesh Neupane, Gurbi Khanal, Mipsang Lama, Gyan Krishna Kayastha

**Affiliations:** 1 Internal Medicine, Patan Academy of Health Sciences, Lalitpur, NPL; 2 Internal Medicine, Universal College of Medical sciences, Bhairahawa, NPL; 3 Internal Medicine, Universal College of Medical Sciences, Bhairahawa, NPL; 4 Internal Medicine, Chitwan Medical College, Bharatpur, NPL

**Keywords:** prognostic value, severity prediction, revised atlanta classification, acute pancreatitis, hematocrit

## Abstract

Background and objective

Acute pancreatitis (AP) is a frequent cause of hospitalization for gastrointestinal issues, with a significant proportion of cases requiring intensive care. Although various scoring systems are available to predict AP severity, they often involve inconvenience and can be time-consuming and expensive. Hematocrit, a simple, cost-effective, readily available hematological test, has been used to predict AP severity. However, its effectiveness has been inconsistent across different studies. In light of this, we aimed to analyze the role of hematocrit levels in determining AP severity.

Methods

We conducted a prospective study at Patan Hospital in Lalitpur, Nepal, from June 8, 2022, to June 27, 2023. Sixty-five AP patients were evaluated to determine the prognostic value of hematocrit at admission. The severity of AP was classified per the Revised Atlanta Classification.

Results

Among the patients, 52 (80%) had mild AP (MAP), five (7.69%) had moderately severe AP (MSAP), and eight (12.31%) had severe AP (SAP). The receiver operating characteristic (ROC) curve for admission hematocrit levels yielded an area under the curve (AUC) of 0.551 (95% CI: 0.423-0.675). A hematocrit cutoff value of 42% resulted in a sensitivity of 69.23% and a specificity of 46.15% for predicting severe AP (MSAP + SAP).

Conclusions

Based on our findings, hematocrit at admission is not a strong predictor of the severity of AP.

## Introduction

Acute pancreatitis (AP) is a significant global health issue, ranking high among gastrointestinal conditions that require hospitalization [1]. In 2019, the age-standardized incidence and mortality rates per 100,000 people with AP were 34.8 and 1.4 globally, 55.3 and 1.8 for South Asia, and 25.9 and 2.5 for Nepal [2]. Over the past two decades, The global incidence and hospitalization rates of AP have been on the rise. While the overall mortality for AP is approximately 1%, it can rise to a staggering 30-40% in hospitalized patients with organ failure or pancreatic necrosis [1,3-5].

AP is generally not a severe condition, with most patients (80-85%) experiencing a mild disease. However, around 20% of patients develop moderate to severe AP [6]. The severity of pancreatitis has been classified based on the Revised Atlanta Classification, as follows: mild acute pancreatitis (MAP) - does not involve organ failure or local or systemic complications; moderately severe acute pancreatitis (MSAP) - characterized by organ failure that resolves within 48 hours, local or systemic complications without persistent organ failure, or both; and severe acute pancreatitis (SAP) - marked by persistent (>48 h) organ failure that can involve either single or multiple organs [7,8,9].

Various scoring systems have been developed to predict the severity of AP, but they often prove to be cumbersome, time-consuming, or expensive. In contrast, hematocrit is a simple, cost-effective, and readily available hematological test that has shown promise in predicting the severity of AP. However, the results from different studies on hematocrit have been inconsistent [10-14]. This study, therefore, holds significant potential for contributing to the understanding of the severity of AP and the potential role of hematocrit levels in its diagnosis.

## Materials and methods

Study design and setting

This was a cross-sectional (prospective, analytical, single hospital-based) study conducted at the Patan Academy of Health Sciences, Nepal, from June 8, 2022, to June 27, 2023. Patients older than 14 years with a diagnosis of AP were included. Patients with chronic liver disease, chronic kidney disease, chronic obstructive pulmonary disease, or hematological malignancies and those unwilling to participate in the study were excluded. The study was conducted after obtaining approval from the Institutional Review Committee (IRC) of PAHS (IRC no: PMM2206031633). Informed written consent was obtained from the participants or their guardians. Participants were informed of the study's details, benefits, and potential risks and were allowed to withdraw without citing any reason; patient confidentiality was ensured, and the data were collected on a structured proforma (Appendices)

Acute pancreatitis diagnosis

AP was diagnosed if two of the following criteria were present: severe, persistent epigastric pain (often radiating to the back); serum lipase or amylase levels three times above normal; or characteristic findings on ultrasound, CT, or MRI [8]. AP patients were classified as mild, moderately severe, or severe based on the Revised Atlanta Classification [8,9].

Sampling and statistical analysis

The sample size calculation was based on the statistical analysis of Obuchowski et al. [15]. In the study conducted by Bohara et al., the area under the ROC curve for hematocrit at admission was 0.713 (95% CI: 0.536 - 0.889); the exact value of the area under the ROC curve was used to calculate the sample size [16]. The sample size calculated using the above formula through easy ROC statistical software was 65: 13 patients and 52 controls. Cases were defined as patients diagnosed with either MSAP or SAP and controls were defined as patients with MAP according to the Revised Atlanta Classification.

The data were entered and analyzed using EPI-INFO. ROC analysis was performed using the software EZR. ROC curves were generated to assess admission hematocrit levels as a predictor of acute pancreatitis severity, with the revised Atlanta Classification serving as the gold standard for severity. Categorical variables (sex, age) are presented as numbers and percentages (%), and continuous variables (length of hospital stay) are presented as the mean ± standard deviation (SD), and median and interquartile range (IQR).

## Results

Sixty-five patients meeting the inclusion criteria were enrolled in the study. Among them, 52 (80%) were diagnosed with MAP, five (7.69%) with MSAP, and eight (12.31%) with SAP. The patients' ages ranged from 21 to 79, with a median age of 40 (95% CI: 38.00-44.87 years). Regarding sex distribution, 56 patients were male, while nine were female. The median duration of hospital stay for AP patients in our study was four days (95% CI: 3-4 days). The median duration of hospital stay for patients with mild, moderately severe, and severe pancreatitis were 3.5 days, 11 days, and 7.5 days, respectively (Table 1).

**Table 1:** Characteristics and findings in the study population AP: acute pancreatitis; MAP: mild acute pancreatitis; MSAP: moderately severe acute pancreatitis; SAP: severe acute pancreatitis

Severity of AP	No. of patients	Sex		Median hematocrit, %	Median duration of hospital stay, days
		Male	Female		
MAP	52	45	7	42	3.5
MSAP	5	4	1	35	11.0
SAP	8	7	1	42	7.5
Total	65	56	9		

**Figure 1:**
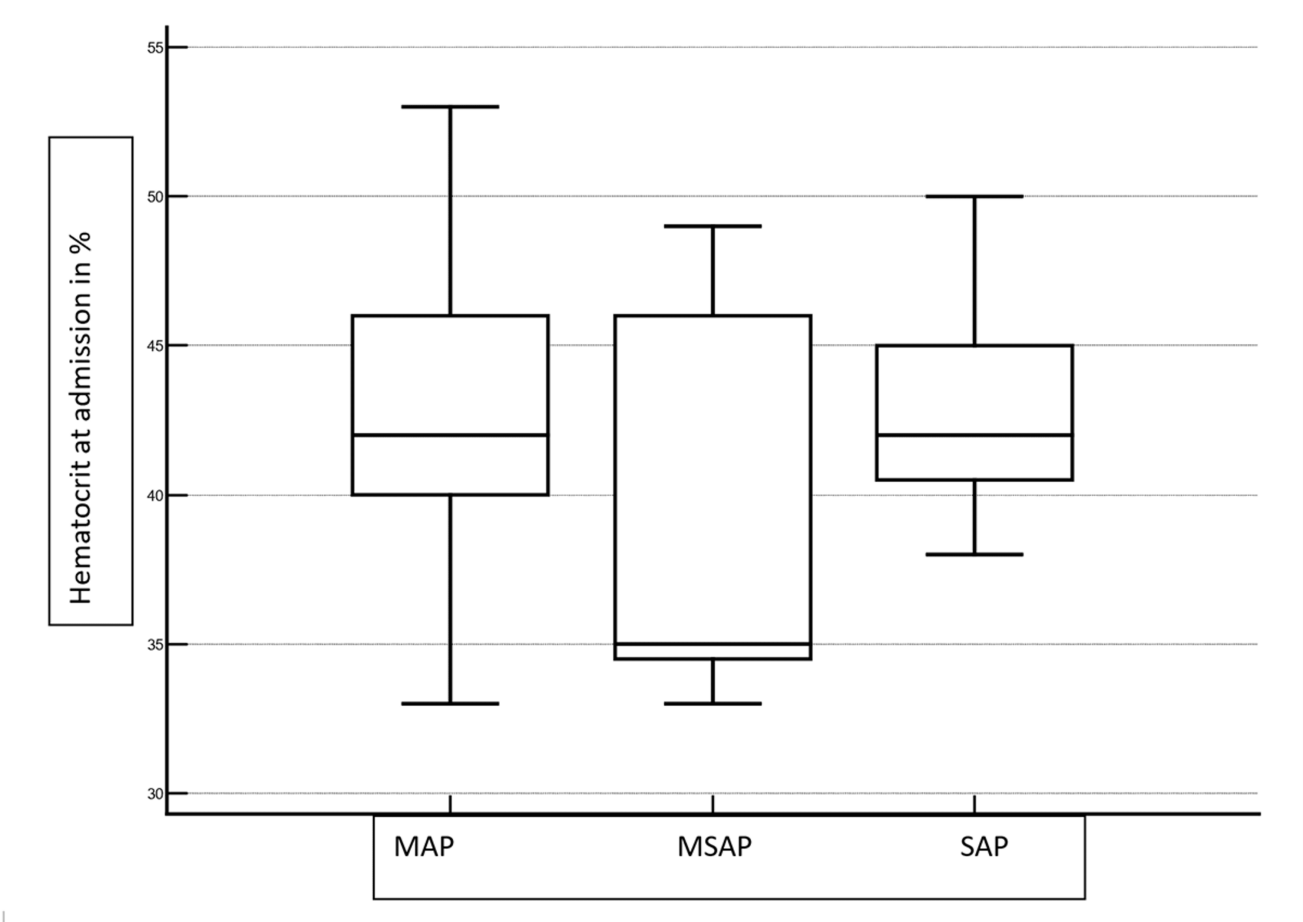
Hematocrit levels with severity of AP AP: acute pancreatitis; MAP: mild acute pancreatitis; MSAP: moderately severe acute pancreatitis; SAP: severe acute pancreatitis

The median hematocrit levels for mild, moderately severe, and severe pancreatitis in the present study were 42%, 35%, and 42%, respectively (Figure 1).

**Figure 2:**
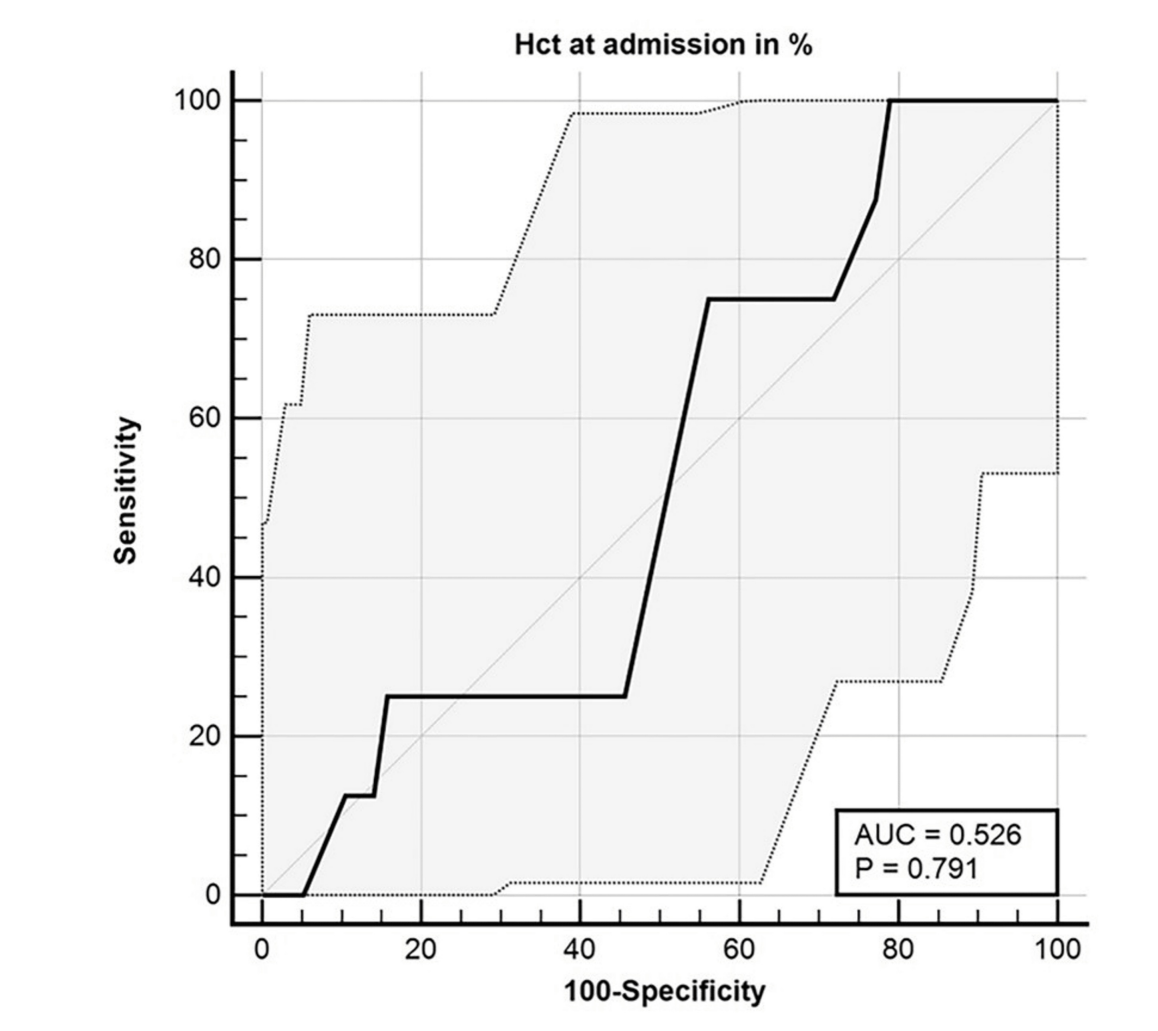
ROC curve for hematocrit at admission as a predictor of SAP AUC: area under the ROC curve; Hct: hematocrit; ROC: receiver operating characteristic; SAP: severe acute pancreatitis

The ROC curve analysis indicated an area of 0.526 for admission hematocrit, with a 95% CI of 0.399 to 0.652. The hematocrit cutoff value was 37%, the sensitivity was 100%, and the specificity was 21.05% for using hematocrit at admission as a predictor of SAP (Figure 2).

**Figure 3:**
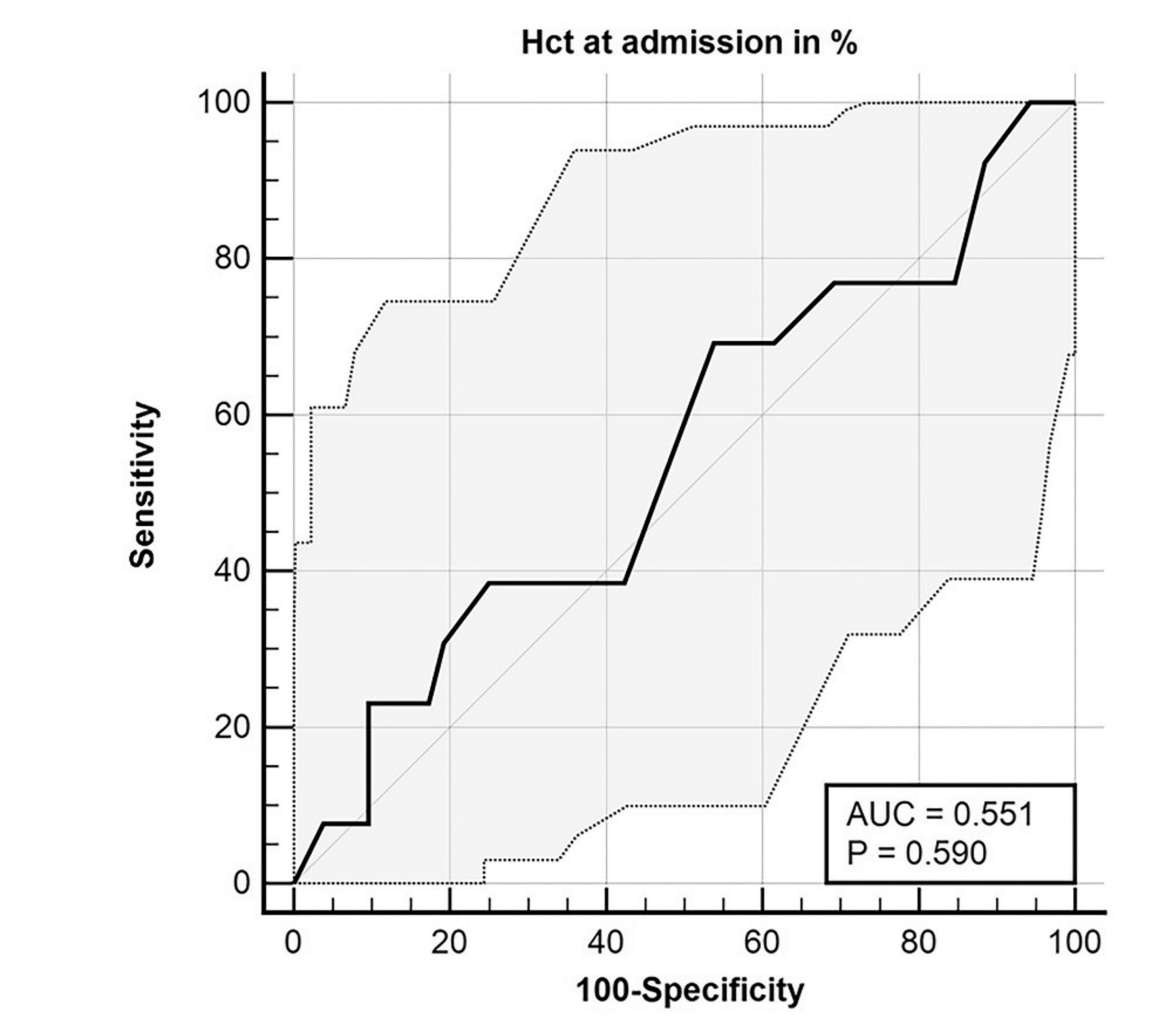
ROC curve for admission hematocrit predicting AP severity (MSAP + SAP) AP: acute pancreatitis; AUC: area under the ROC curve; MSAP: moderately severe acute pancreatitis; ROC: receiver operating characteristic; SAP: severe acute pancreatitis

The ROC curve analysis revealed an area of 0.551 for admission hematocrit, with a 95% CI of 0.423 to 0.675. The hematocrit cutoff value was 42%, the sensitivity was 69.23%, and the specificity was 46.15% for using hematocrit at admission as a predictor of severity of AP (MSAP + SAP) (Figure 3).

## Discussion

In our study, we observed a median age of 40 years among AP patients, with a notable male predominance of 6.22:1. This finding contrasts with the study by Bohara et al., which reported a mean age of 50.16 years and a nearly equal gender distribution (male-to-female ratio: 1.06:1) [16]. Similarly, Bhattarai et al. found a mean age of 44 and a lower male-to-female ratio of 2.3:1 [17]. The male dominance noted in our study and Bhattarai et al.'s work may be attributed to higher alcohol consumption among males in Nepal [18]. In contrast, studies conducted outside Nepal, such as those at the University Hospital of the West Indies and the Mayo Clinic, have reported a female predominance and nearly equal gender distribution, respectively [19,20]. These regional variations suggest that factors such as alcohol consumption significantly influence the demographics of AP.

Our findings indicate that the median hospital stay for AP patients was four days, with a mean stay of 5.14 days. The duration of hospitalization varied by disease severity, as mild cases experienced shorter stays. This is consistent with findings from the Mayo Clinic study, which reported a comparable mean hospital stay, although severe AP cases had longer durations [20]. Conversely, Bhattarai et al. noted a higher mean stay of 12 days, which could be due to differences in patient management or disease severity [17]. A similar trend was observed in a study from the West Indies, indicating that regional healthcare practices, patient severity, and treatment protocols play crucial roles in determining hospital stay length [19].

Our study found that 80% of patients were diagnosed with MAP, with fewer cases of MSAP (7.69%) and SAP (12.31%). These findings align closely with Bhattarai et al., where 72.6% of patients had MAP, 9.7% had MSAP, and 17.7% had SAP [17]. In contrast, Bohara et al. reported a lower prevalence of MAP (48.4%) and a higher proportion of moderately severe cases (38.7%) [16]. The study by Reid et al. from the West Indies observed mild disease in 61.1% of patients, with moderately severe and severe cases comprising 26.7% and 12.2%, respectively [19]. The Mayo Clinic reported an even higher incidence of mild cases (84%) and lower rates of severe cases (3%), reflecting a more favorable patient profile [20].

Regarding the predictive value of hematocrit at admission, our study found that it was not a strong predictor of SAP. The area under the ROC curve (AUC) for hematocrit at admission was lower than that reported by Bohara et al., who found it to be a more reliable predictor of severity in their cohort [16]. Notably, Bohara et al. indicated that hematocrit at 24 hours was a much stronger predictor of severity, a parameter not evaluated in our study [16]. This comparison underscores the variability in the predictive value of hematocrit across different studies, suggesting that other factors may influence its reliability as a predictor of disease severity.

The findings from a post hoc analysis of three large prospective databases indicate that an admission hematocrit level of ≥44% is a significant predictor of persistent organ failure (POF) in patients with SAP, with an AUC of 0.67, a sensitivity of 59.16%, and a specificity of 74.24% [11]. These results align with the association observed between elevated hematocrit levels and adverse outcomes, such as pancreatic necrosis and prolonged hospitalization. The literature notes a range of proposed cutoff values for hematocrit across different studies, with 44% being the most prevalent, highlighting the importance of standardized thresholds for clinical decision-making [21-23]. Furthermore, the emphasis on adequate fluid resuscitation to reduce mortality and promote hematocrit reduction at 48 hours underscores the need for goal-directed therapy in managing AP [24]. While our study focuses solely on admission hematocrit levels, the post hoc analysis suggests that combining admission hematocrit with other severity predictors, such as blood urea nitrogen (BUN), could enhance the accuracy of predicting complications.

Our study offers valuable insights into the demographic characteristics and disease severity of acute pancreatitis in a specific population in Nepal, addressing a notable gap in the existing literature. By comparing our findings with previous studies, such as those by Bohara et al. and Bhattarai et al., we contextualize our results and highlight the influence of regional factors on disease presentation and management. Additionally, our investigation into the role of hematocrit as a predictor of severe acute pancreatitis, despite its limited predictive value, contributes to understanding this parameter and opens new avenues for future research on other predictive factors.

Despite providing these insightful findings, our study has several limitations. The reliance on admission hematocrit alone may overlook rapid disease progression in some patients. Additionally, the single-center study design with a small sample size limits the generalizability of our results. Furthermore, we did not assess changes in hematocrit over time or evaluate morbidity and mortality related to acute pancreatitis, both of which are essential for understanding the full impact of the disease. Addressing these limitations in future research could provide a more comprehensive understanding of acute pancreatitis and its clinical implications.

## Conclusions

Our study showed that hematocrit at admission is not a strong predictor of the severity of acute pancreatitis. However, multicenter studies with large sample sizes are recommended to validate our findings. Studies using admission hematocrit combined with other predictors of the severity of AP would be relevant and provide deeper insights.

## Appendices

Data collection form

Name

Age of the patient

Gender

Hospital number

Date of admission (DD/MM/YYYY)

Date of discharge/death (DD/MM/YYYY)

Duration of hospital stay (days)

Vitals (at first encounter at the emergency department)

a) Blood pressure (systolic/diastolic, mmHg)

b) Respiratory rate (breaths/min)

c) Temperature (°F)

d) Heart rate (beats/min)

e) SpO­_2_ (%)

Laboratory Parameters

a) Total leucocyte count (TLC)

b) Hct at admission (%)

c) Creatinine (mg/dl)

Atrial Blood Gas Analysis (ABG)

a) pH

b) paO_2_

c) FiO_2_
